# Liquid Nitrogen as a Novel Treatment for Gingival Depigmentation: A Case Report

**DOI:** 10.7759/cureus.78099

**Published:** 2025-01-27

**Authors:** Kamala K A, S Sankethguddad

**Affiliations:** 1 Oral Medicine and Radiology, School of Dental Sciences, Krishna Vishwa Vidyapeth Deemed to be University, Karad, IND; 2 Periodontology, HKDET’S Dental College Hospital and Research Institute, Humnabad, IND

**Keywords:** cryosurgery, esthetics, gingival hyperpigmentation, liquid nitrogen, management., novel approach

## Abstract

Gingiva is an important part of a smile, and it is considered the most frequently pigmented tissue in the oral cavity. Although gingival pigmentation is physiologic in most cases, it is one of the common causes of esthetic concerns in patients with gummy smiles. Hence, the demand for cosmetic therapy of gingival melanin pigmentation has become common. Numerous procedures have been suggested to treat this problem, such as gingivectomy, free gingival grafts, cryosurgery, electrosurgery, laser therapy, etc. Here we report a case of gingival hyperpigmentation using liquid nitrogen cryosurgery. The six-month follow-up examination confirmed effective depigmentation with no complications. This simple technique proves to be an excellent choice for managing gingival pigmentation, offering both pleasing cosmetic results and high patient satisfaction.

## Introduction

Gingival hyperpigmentation (GH) is a common physiological condition primarily seen in individuals with darker skin tones [1]. Patients with GH often express concerns about the appearance of their dark-colored gums, particularly as it poses a cosmetic issue for individuals with high smile lines and prominent gingival displays [2,3].

Oral pigmentation can be either physiologic or pathologic in origin. Pathologic pigmentation is categorized into exogenous and endogenous types based on its cause. Exogenous pigmentation may result from factors such as drug use, tobacco or smoking, amalgam tattoos, or heavy metal deposition. Endogenous pigmentation can arise from various factors, including endocrine disorders, syndromes, infections, chronic irritation, or conditions that are reactive or neoplastic in nature [4].

In certain populations, GH is considered a genetic trait independent of age or gender and is therefore referred to as physiologic or racial gingival pigmentation. The extent of melanin pigmentation varies among individuals, primarily due to differences in melanoblastic activity [5]. Several factors contribute to the natural color of the gingiva, like epithelial thickness, degree of keratinization, the number and size of blood vessels, and the amount of pigmentation [6].

The treatment options for GH include gingivectomy, electrosurgery, cryosurgery, bur abrasion, neodymium-doped: yttrium aluminium garnet (Nd: YAG) laser, and the scalpel blade technique [7,8,9]. The selected technique largely relies on the clinician's skill level and individual preference. An ideal treatment modality should ensure minimal patient discomfort, require little to no anesthesia, be operator-friendly, have low technique sensitivity, cause minimal or no bleeding during surgery, allow controlled tissue destruction, result in minimal postoperative complications, and deliver a pleasing, long-lasting outcome [10,11].

Cryosurgery offers a straightforward and affordable method, being relatively low-cost, user-friendly, and free from the strict protocols that are typically related to laser procedures [11]. This method offers several advantages: it is quick and easy to perform, requires no anesthesia or suturing, and causes neither bleeding nor scarring [12,13]. Hence the present case report was carried out to evaluate the clinical efficacy of cryosurgery using liquid nitrogen (LN) in the treatment of gingival melanin hyperpigmentation.

## Case presentation

A 21-year-old female patient reported to the Department of Periodontology, School of Dental Sciences, Krishna Vishwa Vidyapeeth Karad, India, with the chief complaint of black gums that had been present since childhood. As it was an unpleasant look while smiling, the patient needed treatment for the same. The patient’s medical and dental history was noncontributory. On intraoral examination, it was found that the patient had diffuse medium-brown pigmentation in both upper and lower gingiva, which suggested physiological melanin pigmentation. There was no family history of gingival pigmentation. The patient was in good health overall and had good oral hygiene.

The patient was explained about all treatments related to gingival pigmentation and the possibility of repigmentation after a specific period. According to the patient's choice, liquid nitrogen cryotherapy was chosen. A detailed treatment procedure was explained to the patient, and written informed consent was obtained prior to the treatment. Full mouth oral prophylaxis one week prior to the depigmentation procedure was carried out, and the patient was asked to follow oral hygiene instructions.

**Figure 1:**
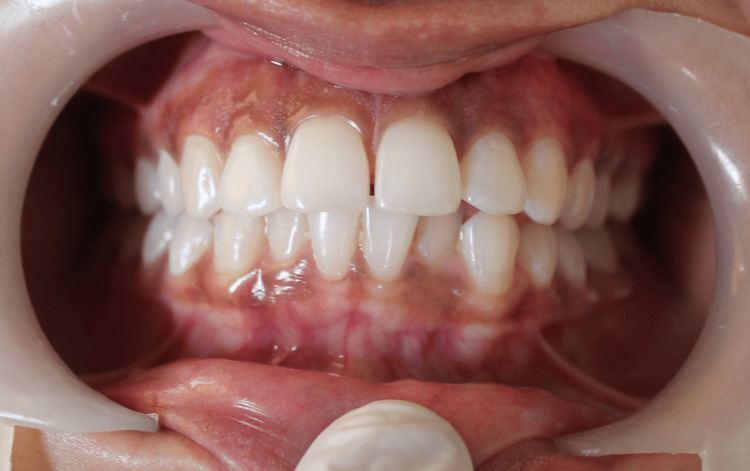
Pre-operative view The image shows gingival hyperpigmentation both in upper and lower anterior teeth region.

An intraoral clinical photograph was taken before the beginning of the procedure. Figure 1 shows diffuse medium-brown hyperpigmentation on both upper and lower gingiva.

The labial gingival area of both the maxillary and mandibular anterior regions extending from tooth number (Universal tooth numbering system) 5-12 and 21-28, respectively, were selected for the depigmentation procedure using liquid nitrogen (LN) (Oxygen Gas Co., Mumbai, India).

**Figure 2:**
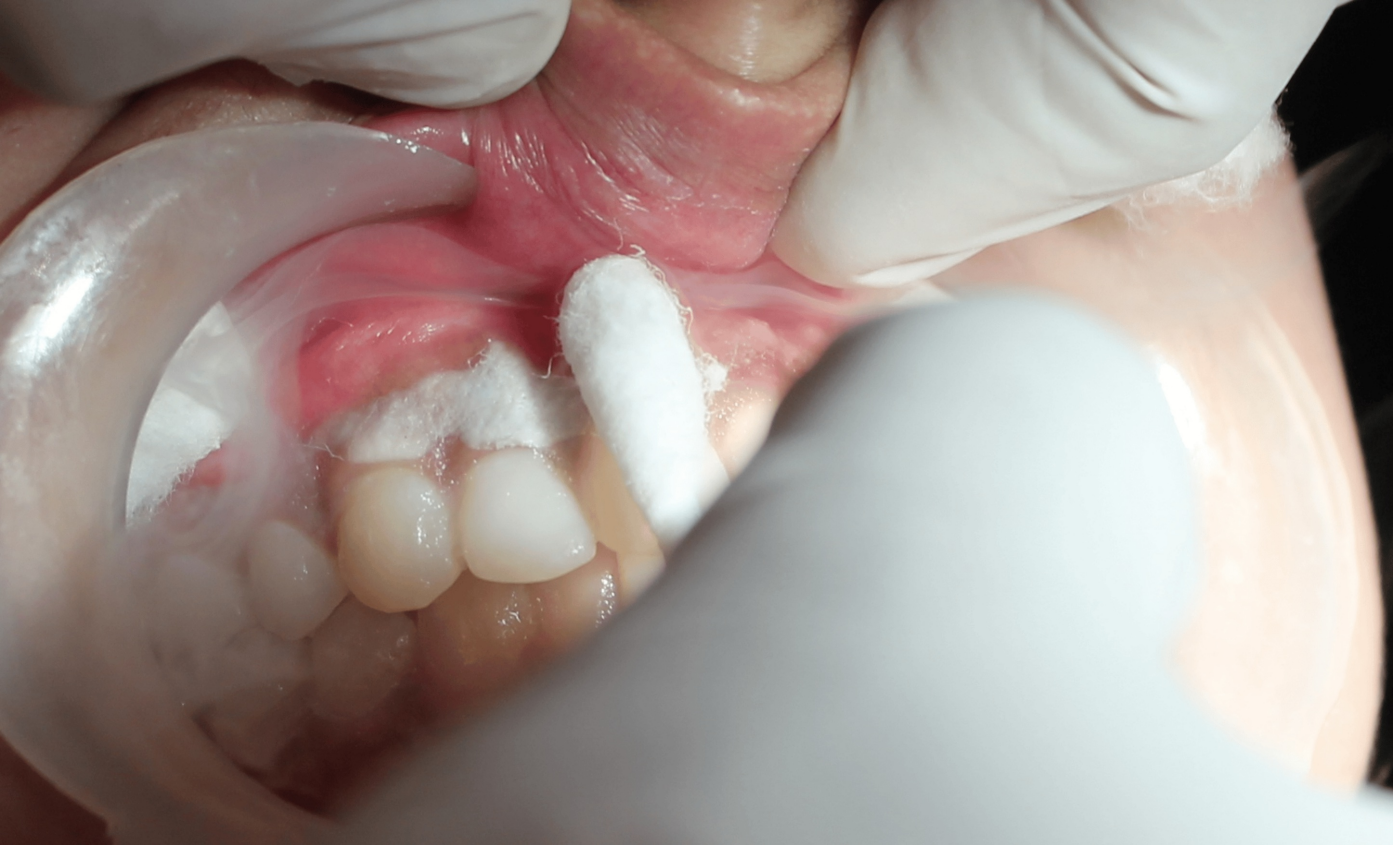
Application of liquid nitrogen (LN) Image shows application of LN with cotton swab in the maxillary arch.

**Figure 3:**
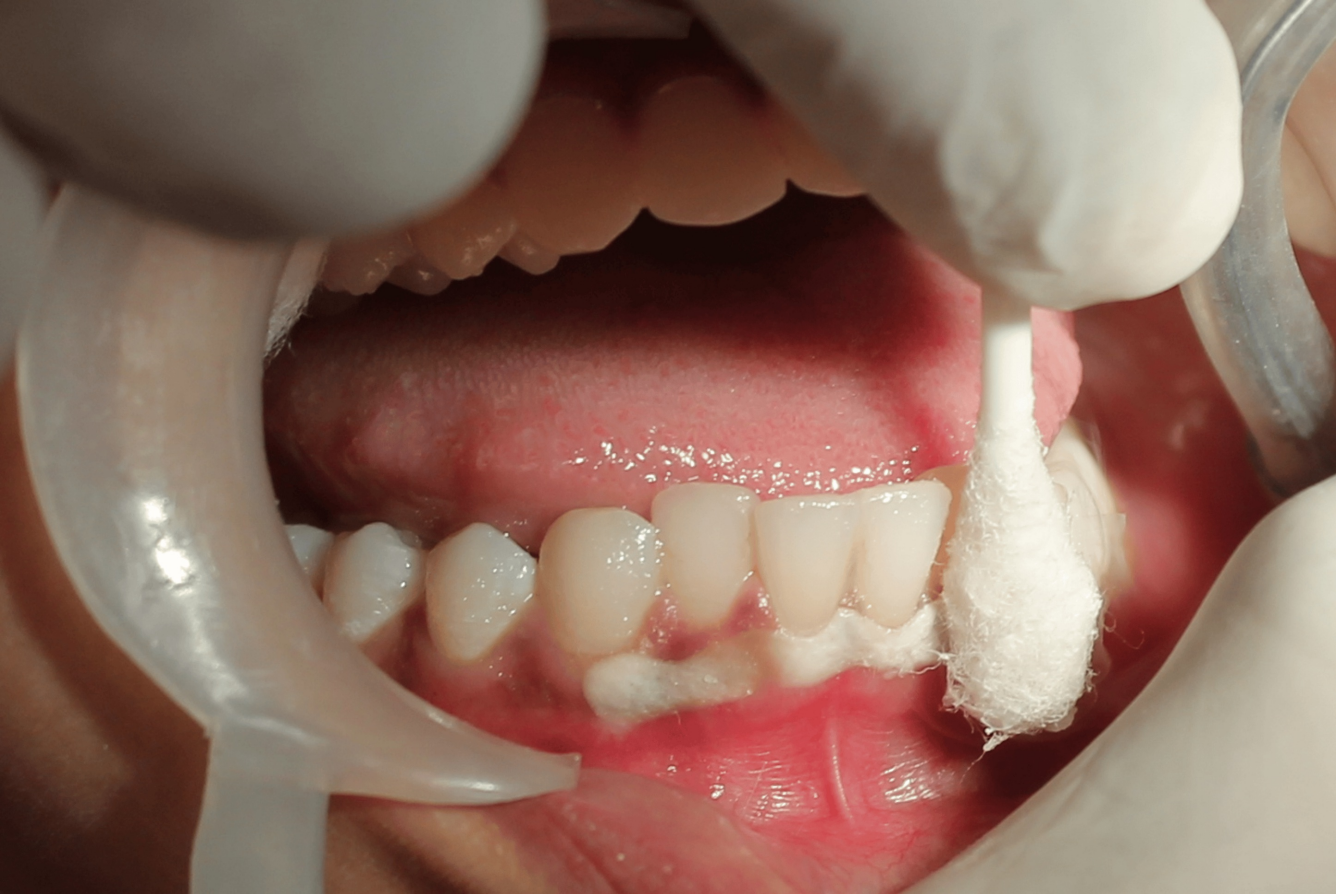
Application of liquid nitrogen (LN) Image shows application of LN with cotton swab in the mandibular arch.

The hyperpigmented gingival areas of treatment in both the maxilla and mandible were isolated using cotton rolls. Lidocaine spray (Lox 10% spray, Neon Laboratories Limited, Mumbai, India) was used to topically anesthetize the area to be treated to minimize the discomfort to the patient. LN was applied sequentially, starting from the right side to the left side in both arches (Figures 2, 3). 

**Figure 4:**
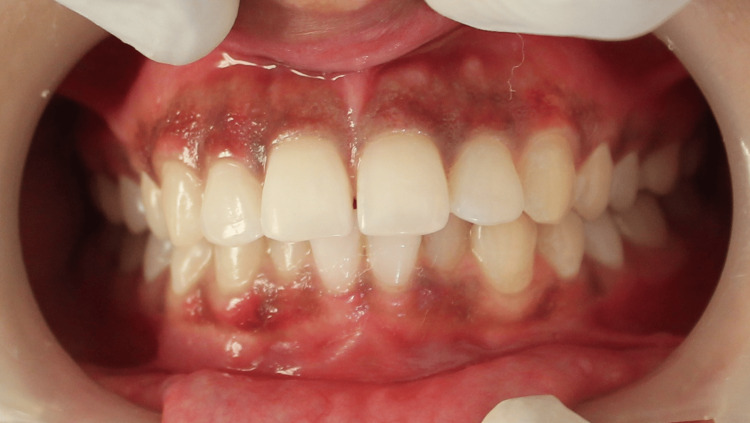
Immediately after the treatment with liquid nitrogen (LN) Image showing slight erythema of gingiva immediately after the procedure.

LN was applied to predetermined areas using a pre-cooled cotton swab (5 mm in diameter) with a rolling motion for 30 seconds until blanching/freezing appearance was observed. Slight erythema of the gingiva was observed immediately after the procedure (Figure 4). The patient was asked to assess the pain using the Numerical Rating Scale, but the patient did not experience any pain except discomfort.

**Figure 5:**
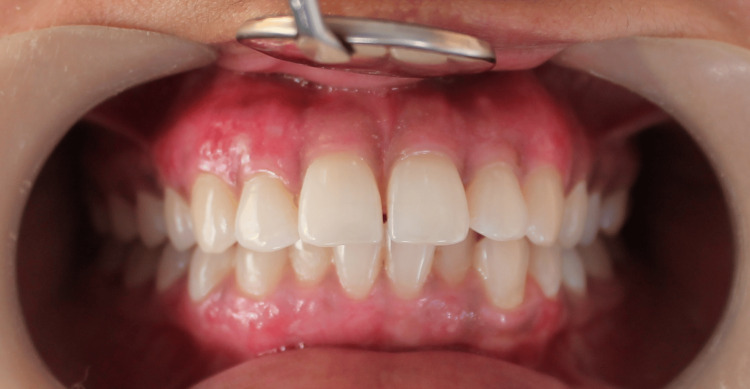
Follow-up of patient after one month Image shows complete healing after one month postoperative treatment.

After the procedure, no periodontal dressing was applied, and the patient was given postoperative instructions regarding oral hygiene maintenance. During postoperative treatment, healing superficial necrosis became apparent, and a whitish layer of slough was formed, and the patient was instructed not to peel off this layer. The gingiva appeared normal, and epithelization was complete within three to four weeks of cryosurgical treatment. The patients did not suffer from any hemorrhage, infection, or scar tissue formation. The initial healing was uneventful following LN application with no postoperative complications. The patient was recalled after one month (Figure 5). The gingiva was pink, firm, and healthy with a distinctive look, and the patient looked satisfied with the aesthetic result.

**Figure 6:**
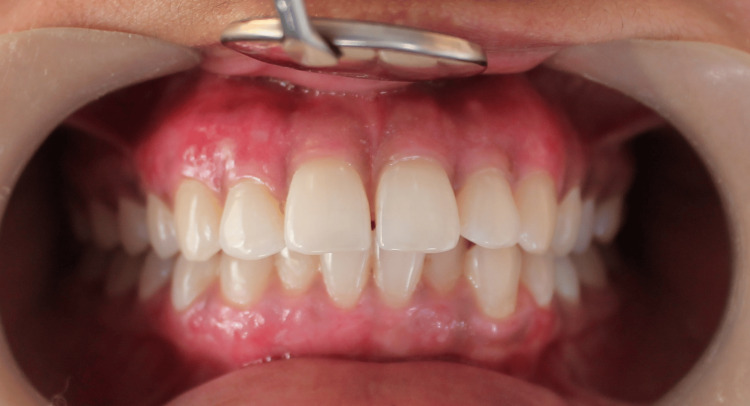
Follow-up of patient after three months Image shows no recurrence of pigmentation after three months follow-up.

**Figure 7:**
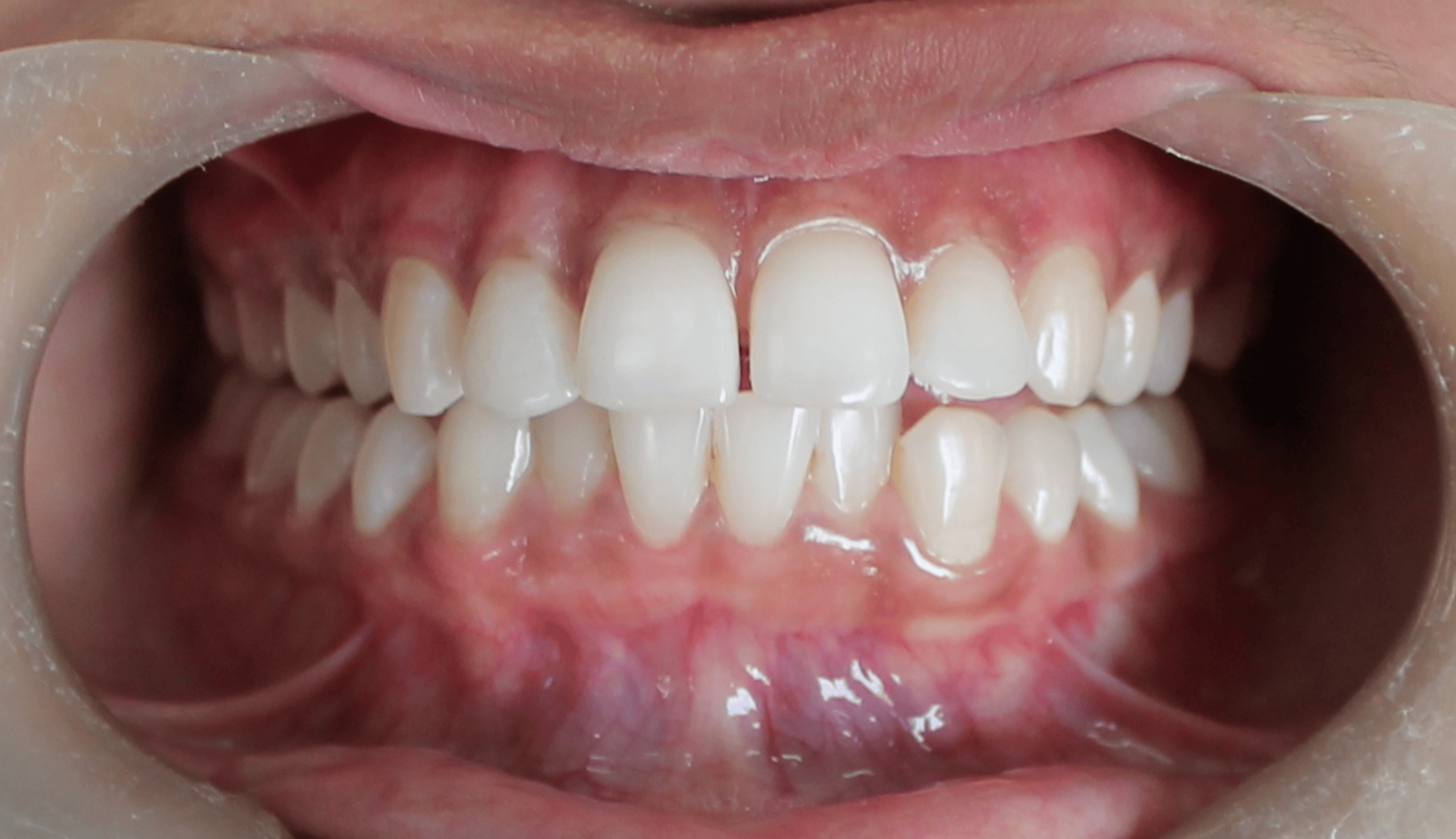
Follow-up of patient after six months Image shows no recurrence of pigmentation after six months follow-up.

The patient was recalled three and six months after the treatment, and no recurrence of melanin pigmentation was noted (Figures 6, 7).

## Discussion

Although melanin pigmentation of the gingiva is completely benign, it can raise cosmetic issues, especially for individuals with a prominent smile line; thus, since the treatment for physiologic pigmentation focuses solely on aesthetics, the ideal method should be painless, safe, and economical [14].

Cryosurgery is one of the most commonly used methods for GH. This technique involves freezing the gingiva with cryogenic agents like LN, which functions at extremely low temperatures. Cryosurgery commonly employs cryogens such as liquid nitrogen, nitrous oxide, and carbon dioxide. The clinical response of oral soft tissues to cryotherapy generally follows a series of distinct stages: tissue swelling (edema), subepithelial hemorrhage, blister formation, necrosis, sloughing of tissue, and, finally, repair [15]. In the current case report, we used liquid nitrogen (-191°C) (Oxygen Gas Co., Mumbai, India) and observed a significant improvement in the gingival color post-treatment, with uneventful healing.

Gingival cryosurgery provides multiple benefits compared to traditional and laser methods. It removes the need for periodontal dressings or sophisticated instruments. Additionally, cryotherapy promotes rapid healing with minimal scarring, delivering excellent cosmetic results. However, a significant challenge of using LN lies in its storage, which necessitates specialized containers or cylinders [14,15]. Wound healing occurs through complete regeneration, supported by sterile inflammatory responses [16]. The use of laser surgery has certain limitations, including a delayed inflammatory response that can cause mild postoperative discomfort lasting one to two weeks. Furthermore, the procedure relies on expensive, specialized equipment, which significantly increases its cost [17,18].

Cryosurgery is a safe procedure that does not necessitate specific safety precautions for the patient, dentist, or assistant. In comparison, laser therapy requires the use of eye protection due to the smoke and vapors generated during the procedure, whereas the vapors produced during cryosurgery are harmless. Furthermore, the direct application of LN carries minimal risk to surrounding tissues, unlike lasers, which may damage the gingiva, enamel, or periosteum. Laser therapy can also cause pain and itching during the first week following treatment and may lead to a loss of tactile feedback during its use [19,20].

Surgical procedures, such as gingivectomy and abrasion, can potentially harm the underlying bone and surrounding tissues, leading to the loss of keratinized tissue and other postoperative complications. Invasive techniques like graft surgery and gingivectomy are associated with various risks, including bleeding, pain, swelling, infection, the necessity for sutures and periodontal dressings, and extended healing periods [13].

In the present study, no repigmentation was observed within six months post-treatment. Similarly, Mokeem reported no recurrence even 18 months after employing the same technique [21]. Previous research comparing liquid nitrogen (LN) and electrocautery demonstrated that LN was superior in terms of wound healing and the pain levels experienced by patients [5,22].

## Conclusions

The findings of this case report suggest that liquid nitrogen (LN) is an effective and cost-efficient method for gingival depigmentation, requiring minimal equipment compared to other techniques. Pain was assessed using the Numerical Rating Scale. In the present case, the patient did not experience pain except discomfort. The procedure caused no pain or bleeding and did not necessitate anesthesia or periodontal dressing. Post-treatment, patients exhibited excellent wound healing, with no recurrence observed during a six-month follow-up.

Clinical evaluations and patient feedback indicate that LN cryotherapy is a simple, safe, painless, and innovative approach for managing gingival melanin pigmentation (GMP). However, further long-term randomized clinical trials with larger sample sizes are needed to confirm these findings.
